# Choosing what works for whom: towards a better use of mechanistic knowledge in clinical practice

**DOI:** 10.1186/s40945-021-00122-1

**Published:** 2021-12-01

**Authors:** Rafael K. Alaiti, Bruno T. Saragiotto, Leandro Fukusawa, Nayra D.A. Rabelo, Anamaria S. de Oliveira

**Affiliations:** 1 Nucleus of Neuroscience and Behavior and Nucleus of Applied Neuroscience, Universidade de São Paulo, São Paulo, Brazil; 2 Research, Technology, and Data Science Office, Grupo Superador, São Paulo, Brazil; 3 Masters and Doctoral Programs in Physical Therapy, Universidade Cidade de São Paulo, São Paulo, Brazil; 4 Institute for Musculoskeletal Health, School of Public Health, University of Sydney, Sydney, Australia; 5 Faculdade de Ciências Médicas Santa Casa de São Paulo, Masters and Doctoral Programs in Health Science, São Paulo, Brazil; 6 Human Motion Analysis Laboratory, Rehabilitation Sciences Department, Universidade Nove de Julho – UNINOVE, São Paulo, SP Brazil; 7 Health Sciences Department, University of São Paulo, School of Medicine of Ribeirao Preto, Ribeirão Preto, SP Brazil

**Keywords:** Chronic pain, Musculoskeletal disorders, Mediators, Mechanisms of action, Decision-making

## Abstract

**Background:**

Clinicians commonly try to use mechanism-based knowledge to make sense of the complexity and uncertainty of chronic pain treatments to create a rationale for their clinical decision-making. Although this seems intuitive, there are some problems with this approach.

**Discussion:**

The widespread use of mechanism-based knowledge in clinical practice can be a source of confusion for clinicians, especially when complex interventions with different proposed mechanisms of action are equally effective. Although the available mechanistic evidence is still of very poor quality, in choosing from various treatment options for people with chronic pain, an approach that correctly incorporates mechanistic reasoning might aid clinical thinking and practice.

**Conclusion:**

By explaining that not all evidence of mechanism is the same and by making a proposal to start using mechanism-based knowledge in clinical practice properly, we hope to help clinicians to incorporate mechanistic reasoning to prioritize and start choosing what may best work for whom.

## Introduction

Modern concepts of pain science point out that chronic pain is a multidimensional experience driven by a complex interaction of various degrees of biological, psychological and social factors. These factors are ultimately associated with large inter-individual variability of symptoms and clinical manifestations [1], making chronic pain very challenging to handle. Clinicians commonly try to use findings from mechanistic studies (e.g., pain neurophysiology) and available conceptual models (e.g., pain-spasm-pain cycle) to make sense of the complexity and uncertainty of chronic pain. They usually find patterns of clinical manifestations to subgroup patients based on signs and symptoms aiming at choosing or justifying their interventions according to the hypothesized mechanisms underlying pain and disability and to the conceptual models adopted [2, 3]. Although this seems intuitive, there are some problems with this approach.

Not all findings of mechanistic studies should be used to explain the interventions’ mechanisms of action or for whom the intervention may work. For example, the results of cross-sectional or case-control studies should not be used to explain how or why an intervention may or may not work. Although clinicians may benefit from using conceptual models to make sense of patient’s complaints and to assist their clinical reasoning, not all frameworks are robust enough to be incorporated in clinical practice. Several frameworks have not been properly investigated yet or does not provide a good explanation of the phenomenon of interest to aid clinical reasoning**.** Therefore, to answer and choose better what works for whom, clinicians will benefit from some guidance on using mechanism-based knowledge in clinical practice. To do that, we need to better understand the mechanisms by which treatments work (i.e., mediators) and for whom it may work (i.e., moderators) while using valid conceptual models to aid clinical reasoning, that for now on will be referred in this text as mechanism-based knowledge.

### When to start using mechanism-based knowledge in clinical practice?

The widespread use of mechanism-based knowledge in clinical practice can be a source of confusion for clinicians, especially when complex classification systems and interventions with very different proposed mechanisms of action are equally effective. For example, although active interventions (e.g., exercise therapy), behavior modification (e.g., graded exposure) and strategies to improve self-management (e.g., graded activity) are effective for people with chronic musculoskeletal pain, all of them produce small effects on pain and disability [4, 5]. Besides that, evidence to date shows that currently available clinical rules and subgroup approaches are not substantially better than applying one general approach [6].

The use of mechanistic reasoning in clinical practice is important. However, it may increase even further the complexity of treatments offered and the number of clinical tests used with no clear indication that it will improve patients‘outcomes [7]. This raises the question:

*“Should we just choose among the available evidence-based intervention based on clinical expertise and patient preferences, whatever their proposed mechanism of action and moderating factors, or should we take the evidence of mechanisms of action and moderating factors into account when deciding among which intervention to choose?”*

While some clinicians choose among different evidence-based interventions based on what they observe during the evaluation process, others might be comfortable to just combine interventions with different proposed mechanisms of action expecting additive effects (even when there is no such evidence). But clinical encounters are brief, and patients usually presents with more than one complaint. Therefore, interventions need to be prioritized and assertive. The available evidence of mechanisms of action and moderating factors for pain interventions is still of very poor quality [8]. However, using an approach that correctly incorporates mechanistic reasoning might aid clinical thinking and practice, or, at best, will minimize confusion until better mechanistic evidence is available.

### Interventions mechanisms of action and moderators of effect

If several effective treatment options with different proposed mechanisms of action are available, clinicians should look for the interventions that maximize the likelihood of improving patient’s symptoms. To do that, clinicians need to understand the mechanisms by which treatments work and for whom it may work (see Table 1). In other words, they should look for the mediators (i.e., a variable by which one intervention affects an outcome [12]) and moderators of effect (i.e., a variable such that the effect of the treatment on the outcome differs for different levels of the moderator variable [9]) studied from randomized clinical trials. This is the only type of evidence that can provide information about the mechanisms by which an intervention works and for whom it may work [12, 13].

**Table 1:** Causal Inference Terms Description for Therapeutic Interventions

Term	Meaning	Example
Intervention’s Effect	Mean between-group difference in a given outcome driven by the intervention [9]	Average pain improvement driven by one intervention (e.g. exercise) in comparison to another (e.g. manual therapy)
Mediators of Effect	A mediator is variables by which one intervention affects an outcome [9]	The effect of exercise on pain is partially channeled through one or several putative mediators (e.g. reduction in pain sensitivity [10])
Moderators of Effect	A moderator is a variable such that the effect of the treatment on the outcome differs for different levels of the moderator variable [9]	High levels of fear-avoidance beliefs are associated with poor treatment outcome in subjects with low back pain [11]

For example, in some cases the effects of two different interventions over an outcome may be mediated by the same causal pathway, e.g., the effect of spine thrust manipulation or mobilization for short-term pain reduction in people with chronic neck pain is mediated by neurophysiological responses [14]. When this is the case and time is scarce, clinicians could freely choose one of the interventions based on its clinical expertise and patient preferences. In other cases, treatment effects may be mediated by multiple mechanisms e.g., cognitive-behavioral approaches may reduce pain and disability by decreasing pain catastrophizing and fear-avoidance [15, 16] while exercises may work by increasing muscle strength [17] and by modulating endogenous opioid mechanisms [10]. In such case, some treatment options will likely to be more effective than others depending on the predominant mechanisms associated with an individual’s pain and could be prioritized instead of others when time is scarce.

When there are known moderators of effect, the individual’s probability of improvement with an intervention may differ according to the different levels of the moderator (e.g., high levels of fear-avoidance beliefs are associated with poor treatment outcome in subjects with low back pain [11]). When this is the case and there are moderators that reduce the probability of improvement, clinicians might benefit by combining multiple interventions that target the same mechanisms for an additive effect, or combine multiple interventions that target multiple mechanisms, trying to increase the probability of treatment success.

In summary, when multiple interventions with different proposed mechanisms of action are equally effective for a given outcome, clinicians may benefit from considering the evidence from mechanisms of action and moderating factors in decision making to improve the patients’ probability of success.

## Call to action

Clinicians are overloaded with the number of available pain conceptual models and evidence showing that complex interventions with very different proposed mechanisms of action are equally effective for chronic pain. This can be misleading and confusing, since the clinical presentation of chronic pain is complex and heterogeneous. We need more research on interventions’ mechanism of action and moderators of effect to explore whether an approach that correctly incorporates mechanistic reasoning can improve clinical thinking and practice. To assist clinicians on how to select good mechanistic evidence to improve their decision making, we described 5 key steps that can be used to identify and interpret high quality mechanistic studies to be applied in clinical practice (Table 2).

**Table:** Table 2 How to identify and interpret high quality mechanistic studies to be applied in clinical practice

1. Use critical thinking and biological plausibility as a starting point to select mechanistic studies. We may place less faith in mediation and moderation analyses that are not consistent with our current understanding of the mechanisms by which a treatment might work or the biological plausibility of a condition. Moreover, mechanistic studies (e.g. cross-sectional study investigating the effect of threat anticipation on motor behaviour) conducted to explore assumptions of a conceptual model or theory (e.g. fear and avoidance model of chronic pain) are not direct applicable in clinical practice and have an exploratory nature. 2. Only relies on mediation and moderation studies conducted from randomized controlled trials. Randomized controlled trials allows the investigation of a temporal sequence between intervention, mediator and outcome, without the influence of confounding variables that may bias the intervention-mediator and intervention-outcome effects. Moreover, the investigation of unbiased treatment moderators of effect is also dependent of the randomization processes. 3. Verify the risk of bias of the randomized controlled trial used to estimate mediators and moderators of effect before interpreting its results. Although randomized controlled trials are the best design to estimate mediators and moderators of effect, trials with high risk of bias are prone to under-estimate or over-estimate the true intervention effect and, therefore, the estimation of the putative mediators and moderators of effect might also be biased. 4. Mediation and moderation analyses must have been planned a priori. Mediation and moderation analyses are secondary analyses and, therefore, should be planned at the protocol stage and be available in the registration or publication of the protocol. Clinicians should read the original study protocol and verify the assumptions used to plan the analysis to avoid reporting bias. 5. Results of mediation and moderation analyses must be interpreted with caution and further validation is necessary. Mediation and moderation analyses that are well-planned and conducted from a trial with low risk of bias can provide useful information for clinical practice and decision making. However, to assume high certainty of evidence that these effects do exist, we suggest seeking for further validation and replication of results in more than one single study.	

Until better mechanistic evidence is available, clinicians should use the available evidence about mechanisms of action and moderating factors to optimize treatment prescription with caution, as we tend to give more credibility to stories that make sense (i.e., a cognitive bias named confidence by coherence). Clinicians should be aware that mechanistic evidence is not a proof of treatment efficacy, no matter how much it makes sense (e.g., hyaluronic acid injections has been shown not to be superior to placebo for hip osteoarthritis, despite existing in vitro evidence show otherwise [18]). Similarly, evidence of efficacy does not necessarily reflect the mechanisms by which the intervention affects the outcome (e.g., motor control exercises for people with low back pain does not work through changes in deep muscles activation patterns [19]). Therefore, clinicians should choose among the available evidence-based treatments and use good conceptual models with the available evidence of mechanisms of action and moderators of effect to choose what may best work for whom. This can also clarify the information given to patients regarding the choice of their treatments, helping them to better understand their condition.

## Conclusion

Current available mechanistic evidence does not support a mechanistic reasoning to clinical decision-making in choosing between various treatment options for people with chronic pain. However, clinicians do use mechanistic evidence (or what they think that are good mechanistic evidence) for clinical reasoning and for justifying their interventions (for themselves and for patients). By explaining that not all evidence of mechanism is the same and by making a proposal to identify, interpret and start using mechanism-based knowledge in clinical practice properly (assuming that in several conditions, the quality of evidence is and will be poor to aid clinical reasoning) we hope to help clinicians to properly incorporates mechanistic reasoning in practice. This may lay the foundation for the future, when higher-quality evidence are available.

## Data Availability

NA, no data
